# Proinflammatory Effects of Diesel Exhaust Nanoparticles on Scleroderma Skin Cells

**DOI:** 10.1155/2014/138751

**Published:** 2014-06-01

**Authors:** A. Mastrofrancesco, M. Alfè, E. Rosato, V. Gargiulo, C. Beatrice, G. Di Blasio, B. Zhang, D. S. Su, M. Picardo, S. Fiorito

**Affiliations:** ^1^Laboratorio di Fisiopatologia Cutanea, Istituto Dermatologico S Gallicano, Via E. Chianesi 53, 00144 Roma, Italy; ^2^Istituto di Ricerche sulla Combustione (IRC), CNR, Piazzale V. Tecchio 80, 80125 Napoli, Italy; ^3^Dipartimento di Medicina Clinica, Università Sapienza, CNR, Viale dell'Università 37, 00185 Roma, Italy; ^4^Istituto Motori (IM), Via Marconi 8, 80125 Napoli, Italy; ^5^Shenyang National Laboratory for Materials Science, Institute of Metal Research, Chinese Academy of Sciences, Shenyang 110016, China

## Abstract

Autoimmune diseases are complex disorders of unknown etiology thought to result from interactions between genetic and environmental factors. We aimed to verify whether environmental pollution from diesel engine exhaust nanoparticulate (DEP) of actually operating vehicles could play a role in the development of a rare immune-mediated disease, systemic sclerosis (SSc), in which the pathogenetic role of environment has been highlighted. The effects of carbon-based nanoparticulate collected at the exhaust of newer (Euro 5) and older (Euro 4) diesel engines on SSc skin keratinocytes and fibroblasts were evaluated *in vitro* by assessing the mRNA expression of inflammatory cytokines (IL-1**α**, IL-6, IL-8, and TNF-**α**) and fibroblast chemical mediators (metalloproteases 2, 3, 7, 9, and 12; collagen types I and III; VEGF). DEP was shown to stimulate cytokine gene expression at a higher extent in SSc keratinocytes versus normal cells. Moreover, the mRNA gene expression of all MMPs, collagen types, and VEGF genes was significantly higher in untreated SSc fibroblasts versus controls. Euro 5 particle exposure increased the mRNA expression of MMP-2, -7, and -9 in SSc fibroblasts in a dose dependent manner and only at the highest concentration in normal cells. We suggest that environmental DEP could trigger the development of SSc acting on genetically hyperreactive cell systems.

## 1. Introduction

Autoimmune diseases are complex disorders of unknown etiology characterized by immune responses to self-antigens and are thought to result from interactions between genetic and environmental factors. Many independent lines of investigation suggest that the environment, acting on genetically susceptible individuals, plays a causative role in the development of such diseases [1–3]. The study of epigenetic mechanisms in the pathogenesis of autoimmune diseases is receiving unprecedented attention. Epigenetic mechanisms control gene expression and are influenced by external stimuli, linking environment and gene function. A variety of environmental agents, such as viral infection, hormones, certain drugs, and pollutants, have been found to influence the development of autoimmune diseases. On the other hand, there is considerable evidence of epigenetic changes, particularly DNA methylation alterations, in diseases like systemic lupus erythematosus, rheumatoid arthritis, or multiple sclerosis [4, 5]. Systemic sclerosis (SSc) is a complex immune-mediated disease of unknown etiology associated with early inflammation, immune dysfunction, and vascular injury, followed by progressive fibrosis of the skin and internal organs. In this disease the cell infiltration correlates with skin thickening, suggesting a relation between inflammation and fibrosis. Fibrosis, the distinguishing pathological hallmark of SSc, is characterized by an excessive connective accumulation and matrix remodeling [6, 7]. SSc is generally divided into two categories based on the extent of skin fibrosis: diffuse cutaneous SSc (dcSSc) and limited cutaneous SSc (lcSSc) [8, 9]. A possible causative role of environmental factors in the pathogenesis of the disease has been suggested and the importance of several occupational factors in the development of SSc such as crystalline silica dust, white spirit, aromatic solvents, chlorinated solvents, trichlorethylene, ketones, and welding fumes has been highlighted [3]. The association between SSc and occupational exposure may be variable according to gender and the risk of SSc appears to be markedly associated with high cumulative exposure [10–12]. Particulate emissions from diesel exhaust engines (DEP) have been associated with a variety of pathological conditions, including fibrosis. Adverse effects of DEP on human health are currently a serious concern and have been shown to include a higher risk for cancer and pulmonary and cardiovascular diseases [13–16]. Diesel exhaust particles are a category of particulate matter (PM) derived from diesel fossil fuels and combustible engines. PM is divided into three major size categories: ultrafine (<0.1 *μ*m), fine (<2.5 *μ*m), and coarse (<10 *μ*m and >2.5 *μ*m). Particles of nanosized dimension, as small aggregates of carbonaceous particles less than 100 nm, constitute the most part of DEP and represent the greatest concern to human health because they remain in the atmosphere for long periods, invade the indoor air environment, and can be breathed most deeply into the lungs where they are likely to be more toxic than coarse particles [13]. In the last years, as a consequence of the increasing awareness of the public of the potential cytotoxicity of soot particulates, as recently confirmed by the World Health Organization (WHO), the legislative body has imposed increasingly more strict emission levels. The modifications made to the diesel engine to fulfill the low emission standard have essentially changed the morphology and surface functionalization of the soot drastically increasing the chemical reactivity of the carbon surface, thus making it more cytotoxic [17, 18]. Like airway epithelial cells, the epidermal cells, as constitutive elements of the outermost barrier in direct contact with air pollutants, are among the cell populations most exposed to chemical pollutants, including DEP. These cells, when stimulated by foreign body materials and/or particles, can activate and release a cascade of proinflammatory and prooxidant mediators which in turn stimulate other cell types, especially fibroblasts, to produce a series of fibrogenic molecules responsible for the development of fibrous tissue [19, 20]. These fundamental functions result from the location of the skin, which is the largest body's organ at the interface between external and internal environment and is devoted to put in place efficient sensory and effector capabilities to differentially react to aggressive stimuli in external environment [21]. In a recent study we demonstrated that DEP are internalised by both monocyte-derived macrophages from peripheral blood and keratinocytes from healthy subject and possess oxidative, profibrotic, and proinflammatory potential [22, 23]. Our previous findings suggest that DEP could have a key role in inducing inflammatory responses and promoting fibrosis in normal human skin. Based on these results, we hypothesized that DEP, by chronically stimulating the release of proinflammatory and profibrotic mediators (cytokines, chemokines, reactive oxygen species, etc.) from skin cells (keratinocytes and fibroblasts), could represent a risk factor for the development of fibrosis in SSc subjects, genetically predisposed to have a dysregulation of the complex cellular inflammatory network [24]. We aimed to investigate whether airborne factors, represented by carbonaceous nanoparticles from both Euro 4 and Euro 5 light duty diesel engines, can stimulate the release of proinflammatory and profibrotic mediators by activated and genetically upregulated keratinocytes and fibroblasts derived from subjects affected with dcSSc and to compare the response* in vitro* to that obtained with cells from healthy subjects.

## 2. Materials and Methods

### 2.1. Particle Collection and Characterization

#### 2.1.1. Experimental Setup

The experimental activities were conducted on a prototype single cylinder research engine which has a modern combustion system design derived from a Euro 5 compliant four-cylinder engine which represents the state of the art of light duty (LD) diesel engine technology.

The engine-out exhaust gases for pollutant and particle analysis were sampled at the same point, upstream of the typical emissions after-treatment systems (diesel oxidation catalyst (DOC) and diesel particulate filter (DPF)). From the same point the exhaust gases were drawn off and collected on a filter where the soot was collected. The gaseous emissions were recorded with an ABB, EMERSON, and Ecophysics device for UHCs, CO, CO_2_, O_2_, and NO_*x*_, respectively. The counting and sizing of particles were performed by means of a differential mobility spectrometer (Cambustion DMS 500) in which measurement principle is based on a deflection of electrically charged particles combined with electrical counting. The measurement range is from 5 to 1000 nm [25].

#### 2.1.2. Test Methodology

The test procedure and points were chosen in order to provide additional experimental information on soot characteristics. The operating points were performed using a Euro 4 and Euro 5 engine calibration (derived from the real four-cylinder engine of equal unit displacement) to ensure the value for practical application in the field of LD engines.

The tests were performed at fixed engine (2000 rpm) speed and load (5-bar brake mean effective pressure (BMEP))

#### 2.1.3. Soot Sampling and Pretreatment

Total particulate was collected from the exhaust pipe by isokinetic sampling. The sampling line comprised a Teflon filter (Millipore, pore diameter 0.45 *μ*m) placed in temperature controlled system (360 K) to avoid gas condensation. The solid particulate collected on the filter was washed with dichloromethane (DCM) in order to remove condensable species and fuel residuals (soluble organic fraction (SOF)). The soluble organic fraction (SOF) was further quantified by drying the DCM extracts. It was found that SOF accounts for 8–10 wt.% of the total particulate in both cases. The carbonaceous solid after DCM extraction (soot) was dried, weighted, and characterized. Soot spectral features and hydrodynamic diameter were evaluated in N-methyl pyrrolidinone (NMP) suspensions obtained by using an ultrasonic bath.

#### 2.1.4. Soot Characterization

The chemical-physical characterization of the soot has been performed on soot washed with DCM in order to probe the soot surface without the interference of physisorbed species (unburned hydrocarbons and tar species). The thermal stability of the samples was evaluated by using thermogravimetric analysis (TGA) performed on a Perkin-Elmer Pyris 1 Thermogravimetric Analyzer in oxidative environment (air, 30 mL min^−1^). Fourier transform infrared (FTIR) spectra were recorded on a Nicolet iS10 spectrometer using the attenuated total reflectance (ATR) method. The hydrodynamic diameter of the carbonaceous materials was measured by using a Malvern Zetasizer Nano ZS instrument on soot suspension in NMP (0.01 mg mL^−1^) [26]. Transmission electron microscopy (TEM) and high resolution transmission electron microscopy (HRTEM) imaging were performed on a FEI Tecnai G2 F20 transmission electron microscope equipped with a field-emission gun. Electronic structure measurements were performed using EELS. Spectra were recorded with the Gatan Imaging Filter (GIF 100) with an energy resolution of 1 eV measured at the full width at half maximum (FWHM) of the zero loss. Several spectra are acquired from different areas (0.028 mm^2^) of each sample. Each spectrum is background-subtracted and corrected for multiple scattering. An average of the spectra is calculated. Acquisition of EELS spectra is done using the diffraction mode in order to reduce the current density of the beam on the sample, without decreasing the signal-to-noise ratio in the acquired spectrum. UV-Vis spectra of soot, suspended in NMP, were acquired on an HP 8453 diode array spectrophotometer. With the soot molecular mass being unknown, the absorption coefficients have been expressed on a mass basis (m^2^/g). The concentration of each suspension was 0.01 mg mL^−1^. For the* in vitro *studies, the Euro 4 and Euro 5 soots were sterilized by heating at 180°C, at a temperature lower than that of the exhaust gas at the collection position (200°C), in order to avoid affecting the particle properties. Then the particles were washed three times in distilled water, suspended in PBS at a stock concentration of 1 mg/mL, and sonicated in a water bath at low intensity for 48 h before the use, in order to obtain a better dispersion of the particles that tend to agglomerate.

### 2.2. Cell Cultures

Primary cultures of keratinocytes and fibroblasts from dcSSc patients, classified according to LeRoy et al. [27, 28], and healthy controls were isolated and grown as previously described in [29]. The study was conducted according to the Declaration of Helsinki Principles. Participants gave their written informed consent.

### 2.3. RNA Extraction and Real Time RT-PCR

Primary cultures of keratinocytes and fibroblasts from healthy and dcSSc subjects were stimulated with Euro 4 and Euro 5 soot particles (30 and 60 *μ*g/mL) for 6 h. These concentrations were selected as the lowest concentration able to affect the examined parameters, without inducing excessive cytotoxicity.

After treatment, cells were washed with PBS and harvested for real time RT-PCR analysis. Total RNA was then isolated by the RNAse kit (Qiagen, Hilden, Germany) according to the manufacturer's instruction. The amount and the quality of mRNA were determined using a *μ*QUANT spectrophotometer (BIOTEK, Instruments Inc., Winooski, VT, USA). According to the manufacturer's protocol 1 *μ*g of template RNA was reverse-transcribed to complementary DNA using RevertAid M-MuLV reverse transcriptase (Fermentas Life Sciences, Hanover, MD, USA). Quantitative real time RT-PCR was performed in a total volume of 15 *μ*L with SYBR Green PCR Master Mix (Bio-Rad, Hercules, CA, USA) and a 200 nM concentration of each primer. Sequences of all primers used are indicated in Table 1. Amplification reactions were performed in triplicate for each sample, and analysis of relative gene expression data, normalized to endogenous control (GAPDH), was calculated applying the 2^−ΔΔ*CT*^ on the real time detection system (iQ5 Bio-Rad) supplied with iCycler IQ5 optical system software version 2.0 (Bio-Rad). Expression of each gene was assessed by at least three independent PCR analyses.

### 2.4. Statistical Analysis

All analyses were performed in at least three independent experiments performed in duplicate. Data were reported as mean ± SD followed by statistical significance (Student's* t*-test for unpaired experiments). *P* value of 0.05 was considered to be statistically significant.

## 3. Results

**Figure 1:**
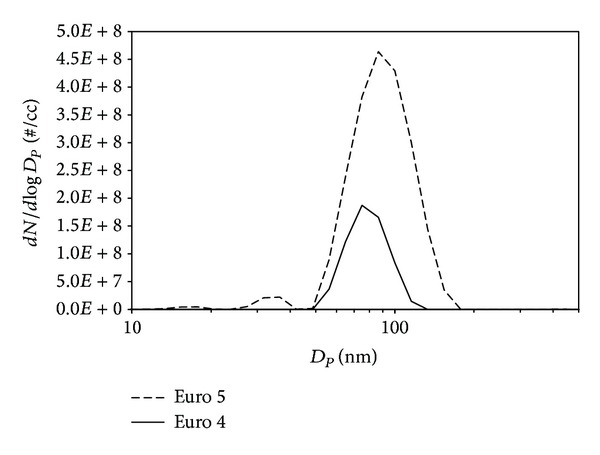
The particle size distribution function (PSDF) of diesel in the range 10–500 nm.

The particle size distribution function (PSDF) of diesel was in the range 10–500 nm (Figure 1). The aerodynamic diameter of soot emitted from Euro 5 configuration appeared slightly larger (90 ± 5 nm) than Euro 4 soot (80 ± 5 nm). This finding was confirmed by the estimation of hydrodynamic diameter measured by DLS (115 ± 5 and 95 ± 5 nm) in NMP suspensions. The soot appeared quite monodisperse, exhibiting a polydispersion index (PI) less than 0.3 in both cases (values of PI < 0.1 are typically obtained for highly homogeneous dispersion, whereas PI > 0.3 for highly heterogeneous dispersions).

**Figure 2:**
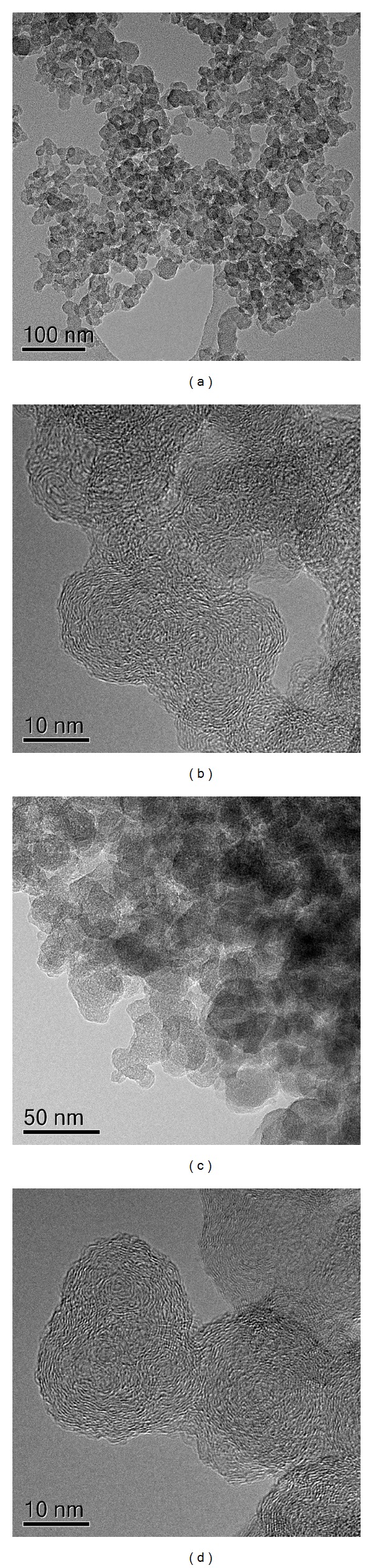
TEM and HRTEM images of the soot particles emitted in Euro 4 (a, b) and Euro 5 (c, d) calibration settings.

TEM and HRTEM images of soot nanoparticles emitted in Euro 4 and Euro 5 calibration settings are depicted in Figure 2. Soot consists of irregularly shaped compact soot aggregates of almost spherical primary particles. The primary particles sizes are very low and keep quite constant (15–20 nm) for both Euro 4 and Euro 5 soots. No variation in dimension is clearly discernible. The interior structure of soot (Figures 2(b) and 2(d)) appears quite similar in both cases, indicating that the different calibration settings negligibly affect the formation of the nuclei cores at the early stage of the soot formation. The irregular soot surface suggests defects in the carbonaceous network arising from the presence of sp3-hybridized carbon in the aromatic moiety.

**Figure 3:**
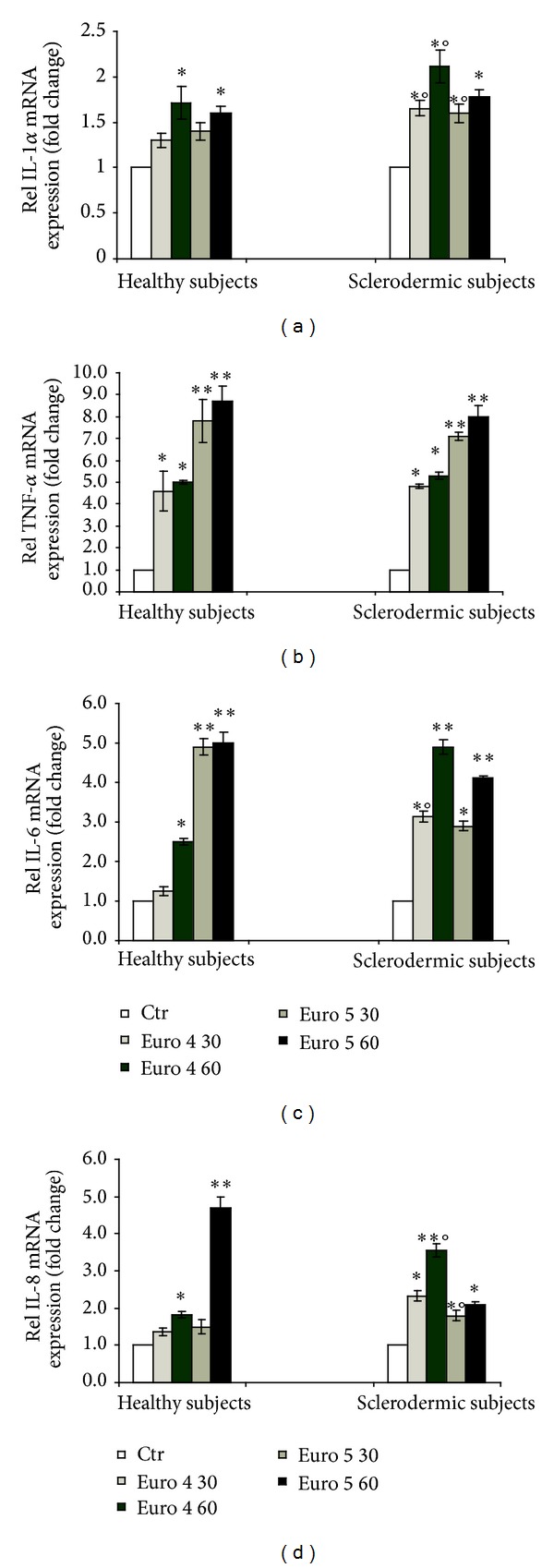
Real time PCR analysis of the expression of IL-1*α* (a), TNF-*α* (b), IL-6 (c), and IL-8 (d) in primary cultures of keratinocytes from sclerodermic and healthy subjects stimulated with Euro 4 and Euro 5 nanoparticles (30 and 60 *μ*g/mL) for 6 h. (*P < 0.01 and **P < 0.001 versus control; °*P* < 0.01 versus healthy subjects).

The impact of the two types of carbonaceous nanoparticles was evaluated on keratinocytes and fibroblasts form healthy and dcSSc subjects. We assessed the proinflammatory potential of Euro 4 and Euro 5 soot particles evaluating their effect on the induction of several proinflammatory cytokine (IL-1*α*, IL-6, IL-8, and TNF-*α*) mRNA expressions by keratinocytes from healthy and sclerodermic subjects. The IL-1*α* and IL-8 mRNA levels were significantly induced in soot particles treated cells from healthy controls only at the highest particle concentration (60 *μ*g/mL) (Figures 3(a) and 3(d)). In dcSSc keratinocytes both soot particles significantly induced the increase of the mRNA level of IL-1*α* and IL-8 cytokines in a dose dependent manner. The IL-6 mRNA expression was significantly induced in Euro 4 treated cells from healthy controls only at the highest concentration, while in Euro 4 treated cells from dcSSc subjects it strongly increased in a dose dependent manner. On the contrary, the Euro 5 treatment significantly induced the IL-6 mRNA expression in a dose dependent manner in both normal and dcSSc cells (Figure 3(c)). Finally, TNF-*α* mRNA expression resulted strongly induced in both cell types treated with Euro 4 and Euro 5 soots (Figure 3(b)).

**Figure 4:**
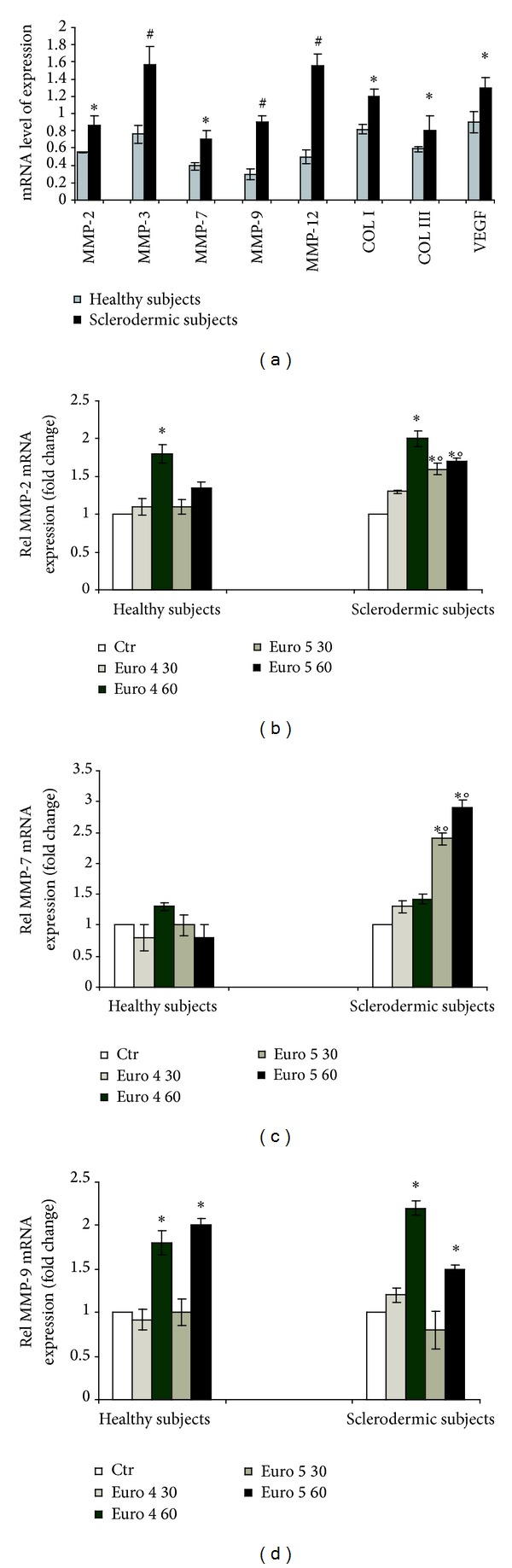
Real time PCR analysis of the expression of MMP-2, MMP-3, MMP-7, MMP-9, and MMP-12, Col I and Col III, and VEGF in primary culture of fibroblasts from sclerodermic and healthy subjects (*P < 0.01; ^#^ *P* < 0.001) (a). Real time PCR analysis of the expression of MMP-2 (b), MMP-7 (c), and MMP-9 (d) in primary culture of fibroblasts from sclerodermic and healthy subjects stimulated with Euro 4 and Euro 5 nanoparticles (30 and 60 *μ*g/mL) for 6 h. (*P < 0.01 versus control; °*P* < 0.01 versus healthy subjects).

We further investigated the effects of these soot nanoparticulates on fibroblast activation, evaluating the mRNA expression of different metalloproteases (MMP-2, MMP-3, MMP-7, MMP-9, and MMP-12), collagen types (Col I and Col III), and vascular endothelial growth factor (VEGF). We found that the mRNA expression of all genes was higher in untreated cells from dcSSc subjects compared with healthy cells (Figure 4(a)). No significant changes in the mRNA expression of MMP-3 and MMP-12, Col I and Col III, and VEGF were observed following treatment with Euro 4 and Euro 5 both in normal and dcSSC cells (data not shown). On the contrary, the mRNA expression of MMP-2 and MMP-9 was induced at the highest dose of Euro 4 (60 *μ*g/mL) in both cell types but was significantly higher in fibroblasts from SSC subjects than in normal fibroblasts (Figures 4(b), 4(c), and 4(d)). The Euro 4 treatment did not induce a significant expression of MMP-7 mRNA level in both cell types (Figure 4(c)). The Euro 5 exposure upregulated the mRNA expression of MMP-2, MMP-7, and MMP-9 in cells from dcSSc subjects in a dose dependent manner (Figures 4(b) and 4(d)). In cells from healthy subjects only the highest dose of Euro 5 induced the expression of MMP-9 mRNA levels.

## 4. Discussion and Conclusions

In the last few years advances in the field of autoimmunity have led to an understanding of the natural history of autoimmune diseases. Autoimmune diseases occur when a genetically susceptible individual encounters an environmental trigger that could be responsible for inducing autoimmunity [30, 31]. Environmental exposures that could play a role in autoimmune disease pathogenesis include infectious agents, ultraviolet light, chemicals and compounds capable of modulating immune responses such as occupational/environmental pollutants or drugs, and behavioral factors, such as smoking and diet [32–36]. Environmental pollution due to nanoparticulate from diesel engine exhaust gases has been hypothesized to be responsible for several bronchopulmonary, cardiac, and vascular diseases. Moreover, numerous epidemiological studies have shown the higher incidence of these diseases in populations living in close proximity to highways, airports, and high density urban traffic zones [37–40]. The present study was performed by challenging* in vitro* skin dcSSc cells with carbonaceous nanoparticles collected from Euro 4 and 5 light duty diesel engines exhaust. Our main purpose was to verify the hypothesis that environmental pollution derived from automotive diesel engine exhaust of actually operating vehicles could have a role in the activation of inflammatory cell pathways in a model of immune-mediated disease. The highly specific characterization and description of particle shape and surface physical-chemical characteristics that have been performed help to better address the influence of one of the multiple constituents of vehicle exhaust emissions in order to make it possible to identify its own effects and to potentially modify its dangerous properties. DEP nanoparticulate consists of irregularly shaped compact aggregates of almost spherical primary particles. The primary particle sizes are very low and keep quite constant (15–20 nm) for both Euro 4 and Euro 5 soots. The very low dimensions of these particles enable them to be easily and spontaneously internalised by skin keratinocytes, as we previously demonstrated [23] and thus to induce a severe inflammatory response by these cells. We observed that DEP nanoparticles activated keratinocytes and fibroblasts of patients with dcSSc at a higher extent as compared to normal cells. In dcSSc keratinocytes both Euro 4 and 5 soot particles significantly induced the increase of IL-1*α* and IL-8 mRNA as well as IL-6 levels even at the lower particle concentration and in a dose dependent manner. A quite different behaviour was observed when cells were treated with Euro 5 particles. In this case the nanoparticle treatment significantly induced IL-6 as well as TNF-*α* mRNA expression in a dose dependent manner in both normal and SSc cells. This cell response could be attributed to the higher proinflammatory potential of Euro 5 with respect to Euro 4 nanoparticles. It can be speculated that a slightly higher graphitic degree of Euro 5 soot, as demonstrated by electron energy loss spectroscopy (EELS) and UV-visible absorption spectroscopy analysis (data not shown), responsible for a different stabilization of radical species on soot surface, could account for this effect. This hypothesis deserves a more focused investigation that is planned in the future.

The higher reactivity of dcSSc keratinocytes, revealed by the increased gene expression of all cytokine evaluated even at the lowest diesel particle concentration, has to be related to the genetically induced upregulation of dcSSc cell inflammatory pathways. Recently, IL-1*α* has been found to be greatly increased in SSc epidermis and it is believed to initiate keratinocyte-fibroblast interactions leading to fibroblast activation. The coculture of the keratinocytes from SSc patients with normal human fibroblasts was found to promote fibroblast activation. A model of chronic injury in SSc in which keratinocyte activation in turn promotes and sustains fibroblast activation and scarring through IL-1*α* has been proposed [41]. Several reports have reported that IL-6 levels are elevated in culture supernatants of dermal fibroblasts and serum from patients with SSc. Its prominent expression in the skin was observed in dermal fibroblasts, mononuclear cells, and endothelial cells in patients with early dcSSc;* in vitro* experiments supported a potent profibrotic effect of IL-6 transsignaling pathways [42–44]. Our findings, demonstrating that DEP induced the increase of IL-1*α* and Il-6 gene expression, both these cytokines possessing a profibrogenic capacity, suggest that this environmental stimulus could represent a key factor capable of initiating a proinflammatory/profibrogenic cascade in SSc subjects. Moreover, these results highlight the fundamental role played by keratinocytes in the development of this disease. In addition, because of the fundamental role played by fibroblasts in the pathogenesis and clinical manifestations of the disease, we evaluated the fibroblast response to DEP treatment. The observation that in untreated dcSSc fibroblasts all metalloproteinases, collagen types I and III, and VEGF genes were overexpressed is in agreement with the literature data and represents the hyperresponsiveness of these cells to unknown stimuli [45, 46]. In the same cells after DEP stimulation, significantly higher levels of MMP-2, MMP-7, and MMP-9 compared to controls were observed. Matrix metalloproteinases (MMPs) constitute a large group of endoproteases that are able not only to cleave all protein components of the extracellular matrix but also to activate or inactivate many other signaling molecules, such as receptors, adhesion molecules, and growth factors. Elevated MMP levels are associated with an increasing number of injuries and disorders, such as cancer, inflammation, and autoimmune diseases. Yet, MMP upregulation has also been implicated in many physiological functions such as embryonic development, wound healing, and angiogenesis and therefore, these proteinases are considered to be crucial mediators in many biological processes [47]. MMP-2, or gelatinase A, a constitutive enzyme, is found in almost all cell types and it degrades denatured collagen (gelatin) and collagen type IV (a component of the basement membrane) as well as other extracellular matrix proteins. MMP-9, or gelatinase B, has been implicated in the pathogenesis of cancer, autoimmune disease, and various pathologic conditions characterized by excessive fibrosis [48, 49]. Dermal fibroblasts from patients with SSc were observed to produce more MMP-9 than those from healthy controls when they were stimulated with IL-1*β*, TNF-*α*, or TGF-*β*. Moreover, the serum MMP-9 concentrations were found to be elevated in SSc patients and to correlate with skin scores. The increased MMP-9 concentrations were attributed to overproduction by dermal fibroblasts [45]. Moreover, serum MMP-9 concentrations were observed to be significantly higher in the diffuse type than the limited type of SSc [50]. SSc is mainly characterized by microvascular damage and excess organ fibrosis. The damage is caused by a massive deposition of collagen and other connective tissue components. In general, tissue fibrosis reflects an imbalance between collagen production and degradation. Excessive accumulation of extracellular matrix (ECM) components, especially types I and III collagen, is the most prominent pathological manifestation of the disease [51, 52]. Numerous studies have shown that an abnormally increased synthesis of these constituents in SSc skin* in vivo* and in cells cultured from the skin of SSc patients grown in tissue culture may be involved in the development of SSc [53, 54]. The observed hyperexpression by dcSSc fibroblasts, before DEP treatment, of metalloproteinase 2, 7, 9, and 12 genes, and their reaction with a further highly significant increase in metalloproteinases 2, 7, and 9 gene expression in response to DEP particles, can be considered as the expression of a tentative, put in place by these cells, to counteract the excessive collagen production in response to inflammatory stimuli. Fibroblast behavior not only confirms that they are hyperreactive to external substances but also demonstrates that diesel nanoparticulate could have a key role in triggering a pathologic response in genetically up-responsive skin cells.

In conclusion, these preliminary results show that environmental factors due to traffic-derived pollution can play a key role in triggering an inflammatory-fibrogenic response by upregulated cells in genetically predisposed individuals that is significantly higher as compared to that of normal cells exposed to the same stimuli.

## Figures and Tables

**Table 1:** Primers used for the real time reverse transcriptase-PCR analysis.

Human	Oligonucleotide sequences (5′-3′)
GAPDH	
Sense:	TGCACCACCAACTGCTTAGC
Antisense:	GGCATGGACTGTGGTCATGAG
MMP-2	
Sense:	TCTCCTGACATTGACCTTGGC
Antisense:	CAAGGTGCTGGCTGAGTAGATC
MMP-3	
Sense:	GCTGCAAGGGGTGAGGACAC
Antisense:	GATGCCAGGAAAGGTTCTGAAGTG
MMP-7	
Sense:	TGAGCTACAGTGGGAACAGG
Antisense:	TCATCGAAGTGAGCATCTCC
MMP-9	
Sense:	TTGACAGCGACAAGAAGTGG
Antisense:	GCCATTCACGTCGTCCTTAT
MMP-12	
Sense:	GAATTGATCCGTTTAGAAGTTTAC
Antisense:	GGCTTGTAGAGCTGTTCAG
VEGF	
Sense:	GTTGACCTTCCTCCATCC
Antisense:	TTCTCTGCCTCCACAATG
Col I	
Sense:	CAGCCGCTTCACCTACAGC
Antisense:	AATCACTGTCTTGCCCCAGG
Col III	
Sense:	TCCAACTGCTCCTACTCGCC
Antisense:	GAGGGCCTGGATCTCCCTT
IL-1*α*	
Sense:	CGCCAATGACTCAGAGGAAGA
Antisense:	AGGGCGTCATTCAGGATGAA
IL-6	
Sense:	AGCCACTCACCTCTTCAGAACG
Antisense:	GGTTCAGGTTGTTTTCTGCCAG
IL-8	
Sense:	CTTGGCAGCCTTCCTGATTTC
Antisense:	TTCTGTGTTGGCGCAGTGTG
TNF-*α*	
Sense:	CAACCTCTTCTGGCTCAA
Antisense:	CGAAGTGGTGGTCTTGTT
